# Optimizing Mouse Surgery with Online Rectal Temperature Monitoring and Preoperative Heat Supply. Effects on Post-Ischemic Acute Kidney Injury

**DOI:** 10.1371/journal.pone.0149489

**Published:** 2016-02-18

**Authors:** Julian A. Marschner, Hannah Schäfer, Alexander Holderied, Hans-Joachim Anders

**Affiliations:** Medizinische Klinik und Poliklinik IV, Klinikum der Universität München, Munich, Germany; Max Delbrueck Center for Molecular Medicine, GERMANY

## Abstract

Body temperature affects outcomes of tissue injury. We hypothesized that online body core temperature recording and selective interventions help to standardize peri-interventional temperature control and the reliability of outcomes in experimental renal ischemia reperfusion injury (IRI). We recorded core temperature in up to seven mice in parallel using a Thermes USB recorder and ret-3-iso rectal probes with three different protocols. Setup A: Heating pad during ischemia time; Setup B: Heating pad from incision to wound closure; Setup C: A ventilated heating chamber before surgery and during ischemia time with surgeries performed on a heating pad. Temperature profile recording displayed significant declines upon installing anesthesia. The profile of the baseline experimental setup A revealed that <1% of the temperature readings were within the target range of 36.5 to 38.5°C. Setup B and C increased the target range readings to 34.6 ± 28.0% and 99.3 ± 1.5%, respectively. Setup C significantly increased S3 tubular necrosis, neutrophil influx, and mRNA expression of kidney injury markers. In addition, using setup C different ischemia times generated a linear correlation with acute tubular necrosis parameters at a low variability, which further correlated with the degree of kidney atrophy 5 weeks after surgery. Changing temperature control setup A to C was equivalent to 10 minutes more ischemia time. We conclude that body temperature drops quickly in mice upon initiating anesthesia. Immediate heat supply, e.g. in a ventilated heating chamber, and online core temperature monitoring can help to standardize and optimize experimental outcomes.

## Introduction

Cooling reduces the cardinal signs of inflammation, i.e. dolor, calor, rubor and tumor, because temperature affects tissue perfusion, oxidative metabolism, and cytokine release. In this context cooling reduces the biological processes of ischemic tissue injury, which provides the rationale for donor hypothermia before kidney transplantation [1], for cooling organs during transport for transplantation [2], and for inducing hypothermia in patients after cardiac arrest [3]. This implies that body temperature is an important determinant for experimental ischemia reperfusion injury (IRI), a widely used method to study the pathomechanisms of acute post-ischemic injury of numerous organs including the kidney [4–6]. Especially mice are susceptible to hypothermia during experimental surgery due to their high surface area-to-mass ratio and a suppression of thermoregulatory mechanisms during anesthesia [7–9]. Insufficient body core temperature control during experimental IRI significantly affects the severity of the injury, i.e. lower temperatures cause less damage [5, 10]. In addition, peri-operative hypothermia is associated with post-operative shivering [11], coagulopathy [12], impaired wound healing, and prolonged anesthetic drug effects [13].

To assure reliable results at low variability any experimental IRI setup must assure a body core temperature in a physiological target range [14, 15]. For this purpose auto-regulated heating plates, heating pads or heating by infrared light are frequently used. Target range body core temperature is rarely validated in each animal throughout the different phases of anesthesia, which explains why duration of ischemia time and corresponding post-ischemic tissue-injury (e.g. serum creatinine) may not be reproducible among different laboratories [16–18].

We hypothesized that an online body core temperature monitoring device would be helpful to validate and improve temperature control in our IRI protocol. We speculated that standard temperature control strategies might be insufficient and report here how online body core temperature monitoring can help to stepwise optimize experimental IRI in a reliable and validated manner.

## Materials and Methods

### Animal experiments

All experimental procedures were carried out according to the German Animal Care and Ethics legislation and were approved by the local governmental authorities, i.e. the Ethical Committee of the “Regierung von Oberbayern”, permit no. (AZ) 55.2-1-54-2532-63-12. Male wild type C57BL/6N mice were purchased from Charles River, Germany. Animals were 6-8 weeks old at the time of experimentation. All mice were housed in groups of five in filter top polypropylene cages in a standard temperature- and humidity-controlled environment with a 12 hours light-dark rhythm and unlimited access to food and water. Cages, nestlets, food, and water were sterilized by autoclaving before use.

### Ischemia reperfusion injury

**Fig 1:**
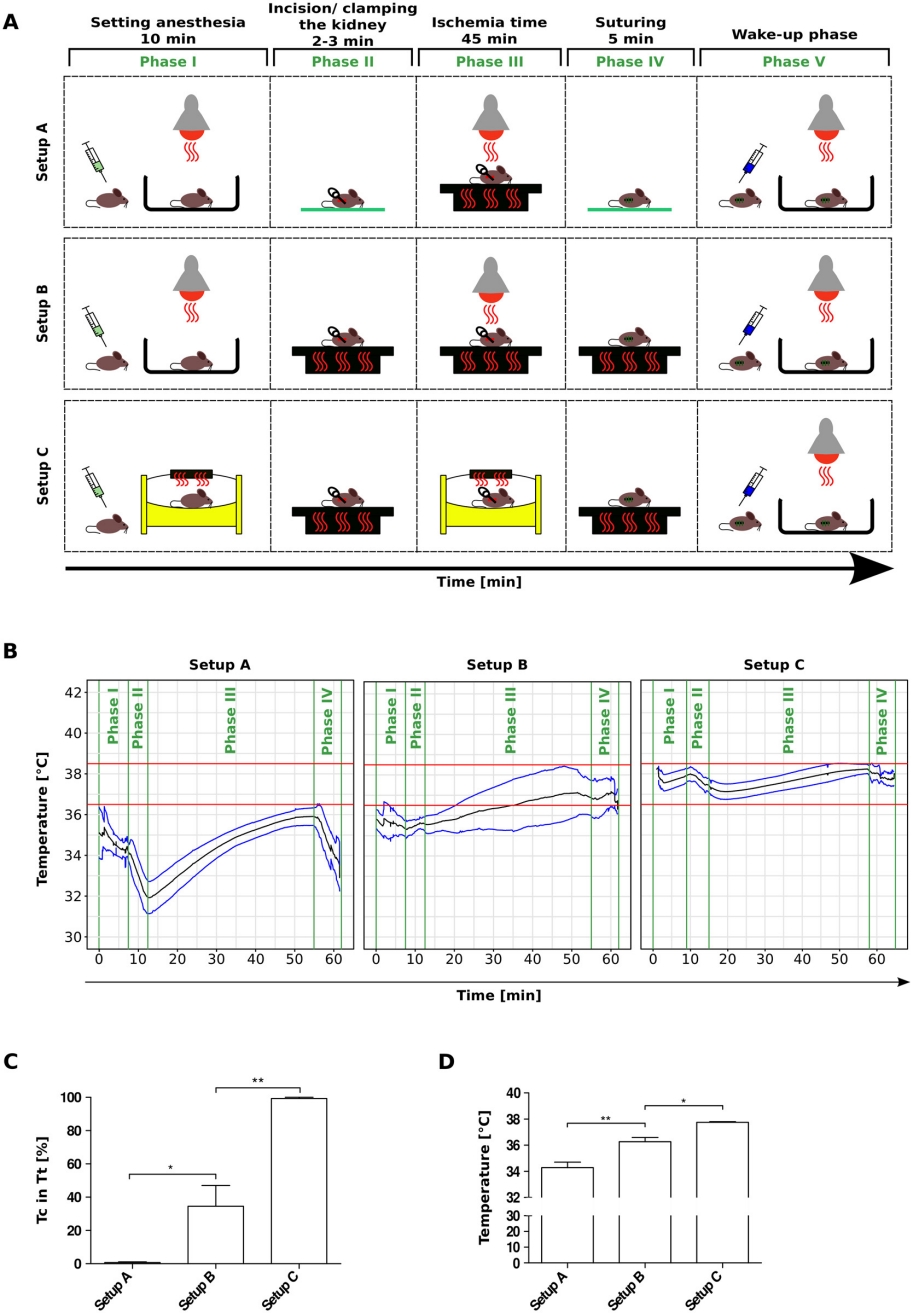
Stepwise optimization of temperature control during experimental IRI. Male C57BL/6N mice, 6–8 weeks of age, underwent unilateral IRI under different temperature control settings. (A) Setup A provides minor heat supply during anesthesia and ischemia time. Setup B extends the former setting by providing heat also during incision/ clamping and suturing via a heating plate. Setup C further replaces the temperature supply elements in the phases of anesthesia and ischemia by an egg breeding device. (B) For each of the setups described in (A) rectal temperature was recorded (ORTR) for each individual mouse, that underwent IRI; the data are presented as mean values (black line) and mean ± SD (blue lines). The temperature target range (Tt) is visualized by two red lines (upper and lower limit). (C) ORTR derived body core temperature (Tc) within the margins of Tt [%] calculated from the data presented in (B), with setup A, B and C giving values of 0.7 ± 1.1%, 34.6 ± 28.0% and 99.3 ± 1.5%, respectively. (D) Mean Tc [°C] (including phase I until phase IV) was calculated from the data presented in (B) with setup A, B and C giving values of 34.3 ± 0.8°C, 36.3 ± 0.7°C, 37.8 ± 0.1°C. Data are expressed as mean ± SEM derived from n ≥ 5.

Mice were anesthetized with a three compound mixture containing 0.5 mg/kg medetomidine, 5 mg/kg midazolam and 0.05 mg/kg fentanyl to achieve analgesia, amnesia and hypnosis prior to sham surgery or unilateral renal pedicle clamping with a micro aneurysm clamp (Medicon, Germany) via a flank incision. Ischemia times applied in this study were 15, 25, 35 and 45 minutes, following reperfusion. Online rectal temperature recording (ORTR) was installed for every mouse after the onset of complete anesthesia. For incision/ clamping and suturing mice were placed on a platform according to the protocol as shown in Fig 1A. After renal pedicle clamping and after clamp removal the successful reperfusion was assessed by color change from pale (ischemia) to the original color [19]. After clamping the kidney was placed back inside the abdomen, with only the clamps handle remaining outside the body. Sham-operated mice were handled in the same manner, except for clamping. After clamp removal wounds were closed using absorbable sutures for peritoneal and cutaneous layer (Ethicon, Belgium). For adjustment of fluid losses, 200 µl of saline were administered into the peritoneal cavity before wound closure. Anesthesia was antagonized using 2.5 mg/kg atipamezole, 0.5 mg/kg flumazenil and 1.2 mg/kg naloxone. 0.05 mg/kg Buprenorphin was injected subcutaneously for pain control. The experiments ended after 24 hours and 5 weeks of reperfusion time, respectively, when the animals were sacrificed by cervical dislocation and kidneys were collected. Post-ischemic and contralateral kidneys were removed, decapsulated, and divided, with one part being stored in 4% buffered formalin, and one part being frozen in RNAlater (Ambion, Germany).

### Heat supply

The respective devices for heat supply during surgery were selected and used in the different phases of the IRI procedure, i.e.: pre-surgical initiation of anesthesia (I), incision and clamping (II), ischemia time (III), suturing (IV) and wake-up phase (V, Fig 1A). In setup A the mice were placed under a infrared lemp during initiation of anesthesia, no heat was supplied during incision, clamping and suturing, but during ischemia time heat was supplied via a heating plate. In setup B additional heat supply was added during incision/ clamping and suturing via a heating plate (Kleintier-OP-Tisch M12511, Medax GmbH, Germany). In setup C heat supply was additionally provided using an egg breeding device (Octagon 20 Advance, Brinsea products Ltd., UK) during initiation of anesthesia and during ischemia time. For the wake-up phase mice were placed under a infrared lamp in all setups. In setup A and B/C the temperature of the heating plate used in phase III and phases II to IV, respectively, was set to 42°C. The sham-operated mice underwent heat supply in setup C. In setup C the final heat supply settings for C57BL/6N male mice with 6–8 weeks of age were set as follows: breeding device phase I 37.0°C, heating plate phase II and IV 42°C, breeding device phase III 39.0°C. Fig 1A shows the setups as described above. ORTR was conducted using Thermes USB temperature data acquisition system and ret-3-iso type T thermocouple probes (Physitemp Instruments Inc., USA).

### RNA extraction, reverse transcription and qRT-PCR

Pure Link RNA Mini Kit (Ambion, Germany) was used to extract total RNA from renal tissue stored in RNAlater, according to the manufacturer’s instructions. cDNA was synthesized from 2 µg of total RNA by reverse transcription polymerase chain reaction (PCR) using Superscript II reverse transcriptase (Thermo Fisher, Germany), 5x first-strand buffer (Thermo Fisher Germany), DTT (Invitrogen, Germany), dNTPs (GE Healthcare, Germany), linear acrylamid (Ambion, Germany), hexanucleotide (Roche, Germany) and RNasin (Promega, Germany). After initial denaturation at 65°C for 10 minutes, reverse transcription was performed at 42°C for 90 minutes using a Mastercycler pro (Eppendorf, Germany).

Quantitative real-time PCR (qRT-PCR) from cDNA was performed using Syber Green (Applied Biosystems, USA), Taq DNA polymerase (New England Biolabs, USA), BioStab PCR optimizer (Bitop, Germany), dNTPs (GE Healthcare, Germany), MgCl_2_ (Fermentas, Germany), and target gene-specific primers (Table 1). The reactions were performed using a Light Cycler 480 (Roche, Germany). 18s rRNA was used as a reference transcript for relative quantification, hence qRT-PCR data for genes of interest are normalized to 18s. Controls consisting of ddH_2_O were negative for targets and reference genes. Each amplification step included an initiation phase at 95°C, annealing phase at 60°C and amplification phase at 72°C, and was repeated for 40 cycles. Primers were designed to be cDNA specific and to target most CCDS approved transcripts. All samples that did not exceed background fluorescence (crossing point/ quantification cycle) of 35 cycles during the amplification reaction were considered not detectable. The melting curve profiles were analyzed for every sample to detect unspecific products and primer dimers. In part, products were visualized on agarose gels.

**Table 1:** Primer pairs used in this study.

Target	Accession no	Forward primer	Reverse primer	efficiency
*18s*	NM 013693.3	GCAATTATTCCCCATGAACG	AGGGCCTCACTAAACCATCC	1.89
*Cxcl-2*	NM 009140.2	CGGTCAAAAAGTTTGCCTTG	TCCAGGTCAGTTAGCCTTGC	1.98
*Il-6*	NM 013541.1	TGATGCACTTGCAGAAAACA	ACCAGAGGAAATTTTCAATAGGC	1.97
*Kim-1*	NM 031168.1	TCAGCTCGGGAATGCACAA	TGGTTGCCTTCCGTGTCTCT	1.96
*Mcp-1*	NM 001166632.1	CCTGCTGTTCACAGTTGCC	ATTGGGATCATCTTGCTGGT	2.01
*Ngal*	NM 011333.3	AATGTCACCTCCATCCTGGT	ATTTCCCAGAGTGAACTGGC	2.03
*Tnfα*	NM 008491.1	ATGGGCTACAGGCTTGTCACTC	CTCTTCTGCCTGCTGCACTTTG	2.25

*18s*: 18s ribosomal RNA, *Cxcl-2*: chemokine-ligand-2, *Il-6*: Interleukin-6, *Kim-1*: Kidney injury molecule-1, *Mcp-1*: monocyte chemoattractant protein-1, *Ngal*: neutrophile gelatinase-associated lipocalin, *Tnfα*: tumor necrosis factor.

### Histological evaluation

Post-ischemic and contralateral kidneys stored in 4% buffered formalin were embedded in paraffin and 2–4 µm sections were prepared for periodic acid–Schiff (PAS) staining and immunostaining, which was performed as described elsewhere [20]. Post-ischemic tubular injury was scored by assessing the percentage of tubules in the outer stripe of outer medulla (OSOM) that displayed cell necrosis in a semi-quantitative manner. For each kidney 15 high power fields (100x) were analyzed. All assessments were performed by a blinded observer. Neutrophils were detected by immunostaining using rat anti-mouse Ly-6B.2 (Serotec, UK). Low power field (50x) images were taken and the amount of Ly-6B.2 positive events was measured using Image J [21] and normalized to whole kidney section area. Renal fibrosis was visualized by Masson’s trichrome staining.

### Statistical analysis

Shaprio-Wilk test for normal distribution, Grubbs’ test for outliers and Levene’s test for homoscedasticy were applied prior to any further analysis. Normally distributed and homoscedastic data were further analyzed using one-way ANOVA and subsequent Tukeys HSD test. In case of heteroscedasticy we applied a Games-Howell test. When distributions deviated from normality we used the Kruskal-Wallis test and the Nemenyi post-hoc test. As far as only two groups were compared (contralateral and ischemic kidney), Student’s t-test was used for normally distributed data. A p-value less than 0.05 indicated statistical significance (shown as n.s. = not significant, * p<0.05, ** p<0.01 and *** p<0.001). Differences compared to the control group (i.e. sham-operated mice or contralateral kidney) are displayed with asteriks (*), whereas differences between treatment groups (i.e. different setups or ischemia times) are displayed with hash (#). Data preparation, evaluation and statistical analysis were performed using R Statistics [22].

## Results

### Heating during ischemia time alone does not control core body temperature

As core body temperature is a critical determinant of post-ischemic tissue injury we established the use of an online rectal temperature recording (ORTR) device to monitor body core temperature profiles during experimental IRI of the kidney using different protocols. The baseline protocol (setup A) is demonstrated in Fig 1A. With this setup all mice showed a significant drop in the core temperature immediately upon anesthesia as well as during the 2–3 minute surgical phase when the clamp has been placed (Fig 1B). During the ischemia time the mice were placed on a heating pad, which gradually increased the core temperature but hardly ever reached the target range of 36.5 to 38.5°C (Fig 1B). This was also the case after clamp removal and wound closure, which was as well associated with a decline of the body core temperature (Fig 1B). During the entire anesthesia only 0.7 ± 1.1% of all temperature recordings met the target range with an average body temperature of 34.4 ± 1.3°C (Fig 1C and 1D). The data indicated that using heating pad during ischemia time alone is not sufficient to control body core temperature during experimental kidney IR.

### Heating from beginning to end of surgery only partially improves core body temperature control

To improve temperature control we extended external heating also during the phase of surgical interventions. Extended use of infrared light has been proven not to be suitable, since the colored light can dampen the vision during surgery. Therefore, we decided to carry out setup B, where the incision and placement of the clamp on the renal pedicle as well as the removal of the clamp and the wound closure were performed on the heating pad (Fig 1A). These modifications prevented the drop in body core temperature during both procedures compared to setup A (Fig 1B). For setup B, 34.6 ± 28.0% of the temperature recordings were within the target range between 36.5 and 38.5°C (Fig 1B and 1C). However, inter-individual variance was not reduced by setup B as illustrated by an average body temperature of 36.3 ± 1.0°C (Fig 1D). However, a significant drop of body temperature was still noted before surgery immediately after installing the anesthesia, which recovered only slowly (Fig 1B). Together, extending the use of heating pad to the surgical procedures partially improved the control of body core temperature within the target range.

### Pre-operative use of a ventilated heating chamber optimizes body temperature profiles

**Fig 2:**
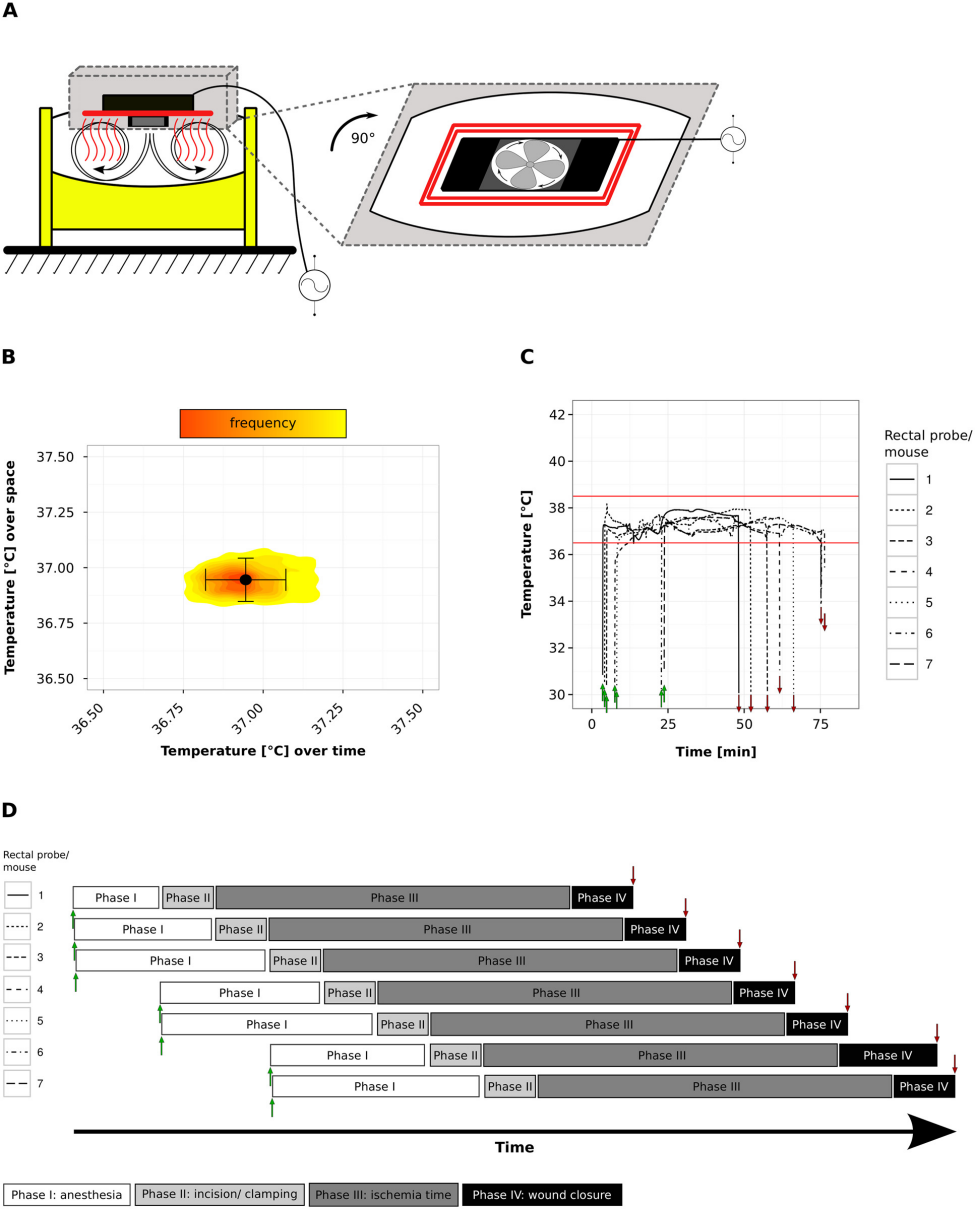
A ventilated heating chamber optimizes body temperature profiles and facilitates the surgery’s work-flow. Schematic representation and temperature profile of the Octagon 20 Advance heating chamber (A and B): The Octagon 20 Advance (A) consists of a solid bottom (yellow) and a transparent upper part, which allows visual control during phase I and II of surgery. The electrical temperature control (black) and the heating element (red) are mounted on top of the chamber. The generated heat is distributed by a small ventilator. (B) The Octagon 20 Advance was set to 37.0°C and continuous temperature recording were conducted for 1 hour at 7 positions inside the breeding device. Temperatures recorded over time and space are plotted on the x- and y-axis, respectively. The black dot represents the overall mean value, the horizontal error bar the standard deviation over time and the vertical error bar the standard deviation over space. The heat-map in the background displays the distribution pattern of recorded temperature values in time and space. Setup C allows parallel surgery in up to 7 mice (C and D): Panel (C) displays a complete, representative set of ORTR generated raw data from 7 mice, where the green arrows indicate the insertion of the probe into the mouse (start of phase I) and where red arrows indicate the end of surgery (end of phase IV). Panel (D) illustrates the work-flow for the 7 parallel surgeries shown in (C). During phase III (ischemia time) of mouse no. 1 a single trained person is able to complete phase II for mice no. 2–7. Anesthetizing 2–3 mice simultaneously in phase I assured a sufficient scope of action for maintaining a constant work-flow, as sequence of surgery can be adapted to the individual body core temperature of every single mouse, which was standardized to be 37.5°C before staring phase II. Therefore, durations of phase I vary. In this specific example phase IV of mouse no. 6 was prolonged by issues during wound closure, which lead to the discontinuity of the steady sequence in surgery work-flow.

To avoid a drop in body temperature during the phase of anesthesia induction we considered a heating chamber, which would allow the animal to move until losing consciousness. The Octagon 20 Advance (Brinsea Products Ltd.) is a ventilated egg breeding device built to keep a uniform distribution of air at a constant temperature (variability < 0.5°C, Fig 2A and 2B). In setup C mice were placed into the first chamber immediately after injection of the anesthetics (phase I) and in a second chamber immediately after replacing the clamped renal pedicle inside the abdomen (phase III, Fig 1A). Each chamber has a capacity for up to seven anesthetized mice, hence, a single trained person can perform the entire interventional procedure in a serial manner, with up to seven mice kept simultaneously in the chamber during ischemia time (Fig 2D), and with each mouse being under online temperature monitoring (Fig 2C). Using setup C the initial drop in body core temperature could be prevented resulting in 99.3 ± 1.5% of all temperature recordings that were within the target range (Fig 1B and 1C) and in a stable body core temperature of 37.7 ± 0.1°C (Fig 1D). With this setup it is possible to sequentially process up to seven mice within 75 minutes at an ischemia time of 35 minutes each and to maintain each mouse within the target range of body temperature (Figs 1B/C and 2C/D). The data have shown that using a ventilated heating chamber, a heating pad, and online body core temperature monitoring can optimize temperature control during experimental kidney IRI.

### Temperature control during experimental ischemia-reperfusion affects kidney injury

**Fig 3:**
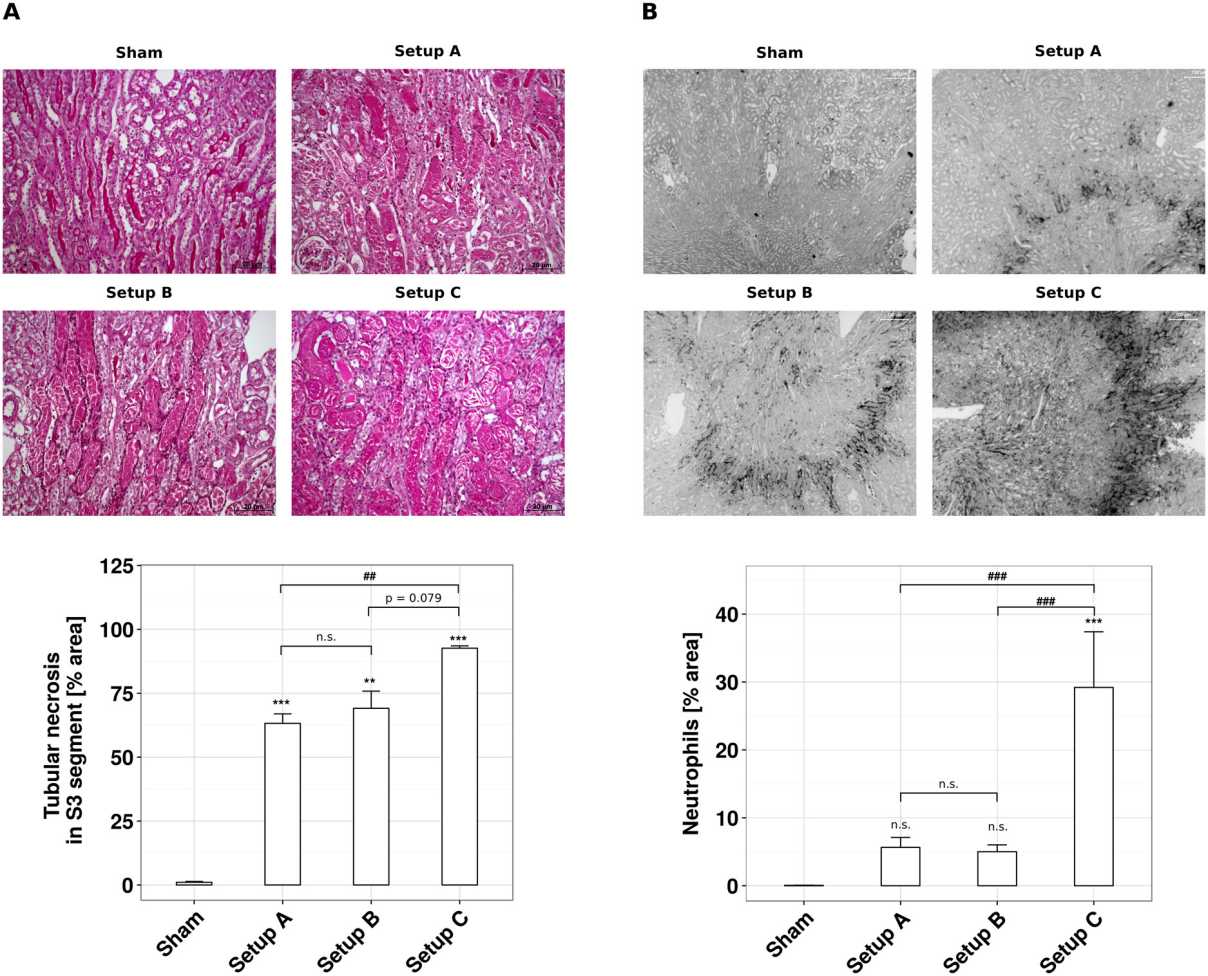
Optimization of temperature control affects tubular necrosis and neutrophil infiltration. Male C57BL/6N mice, 6–8 weeks of age, underwent unilateral ischemia for 45 minutes and subsequent reperfusion for 24 hours using setup A, B and C, respectively. (A) Representative PAS-stained pictures of sham-operated and ischemic kidney sections are shown in 100-fold magnification. The necrotic area of the S3 segment [%] is presented as mean ± SEM, derived from n = 5. (B) Representative Ly-6B.2-stained pictures of sham-operated and ischemic kidney sections are shown in 20-fold magnification. Ly-6B.2-positive events are assessed from 50-fold magnified images and normalized to the entire kidney section area, expressed as mean ± SEM, derived from n = 5.

**Fig 4:**
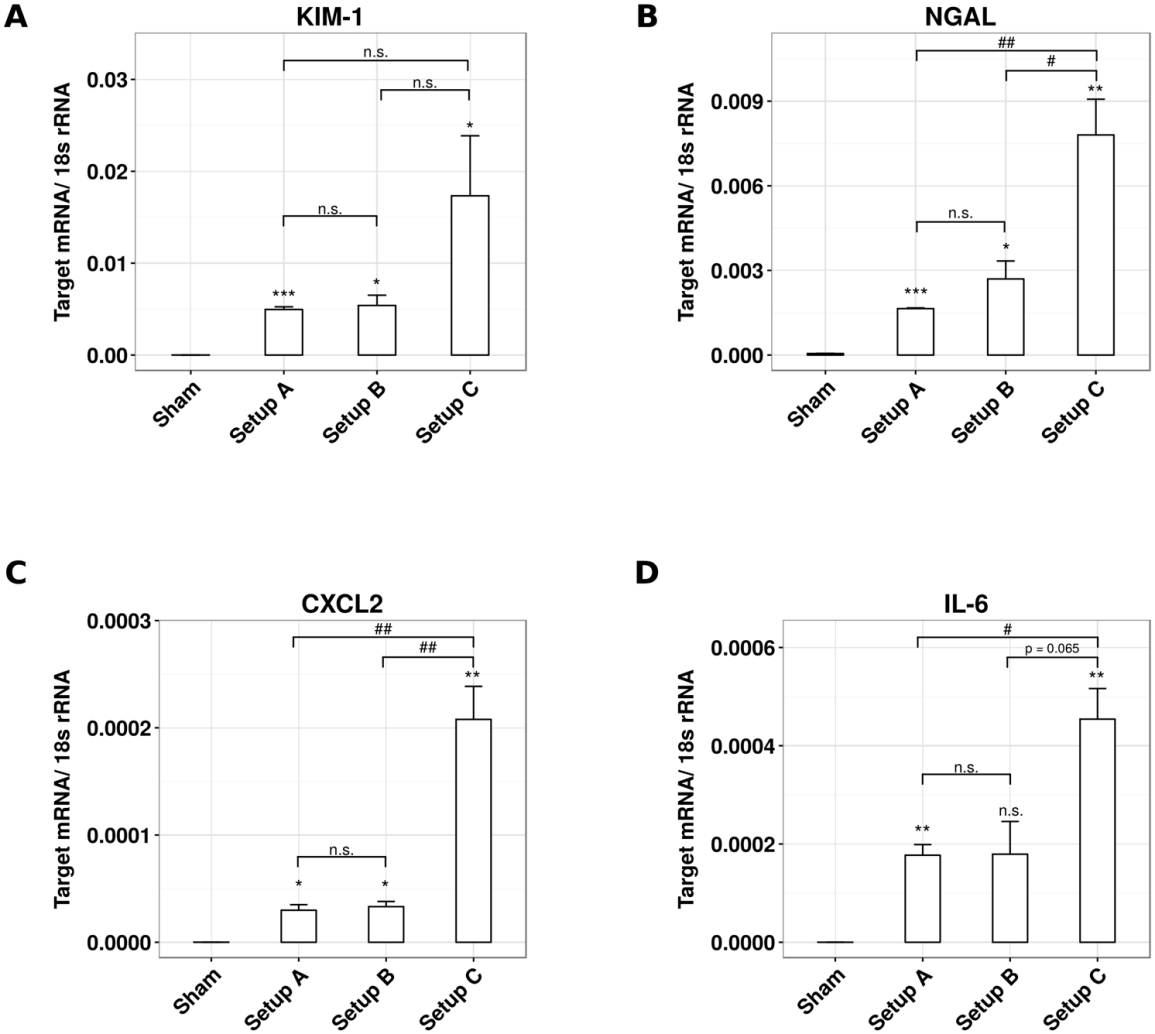
Optimization of temperature control affects mRNA expression levels of pro-inflammatory and kidney injury markers. Male C57BL/6N mice, 6–8 weeks of age, underwent unilateral ischemia for 45 minutes and subsequent reperfusion for 24 hours using setup A, B and C, respectively. mRNA expression levels were assessed by reverse transcription and subsequent qRT-PCR for (A) *Kim-1*, (B) *Ngal*, (C) *Cxcl-2* and (D) *Il-6*. Data are calculated as target gene expression normalized to the housekeeping gene 18s and presented as mean ± SEM, derived from n ≥ 5.

How does validated body core temperature control affect post-ischemic kidney injury? To address this question we analyzed kidney injury after 24 hours of reperfusion by standard histology, immunostaining, and qRT-PCR. With setup A 60% of the tubules of the S3 segment were necrotic (Fig 3A). While temperature control with setup B had no significant effect on tubule injury, setup C significantly increased the percentage of necrotic S3 segment tubules as compared to setup A (Fig 3A). This was consistent with an increased mRNA expression of the kidney injury markers KIM-1 and NGAL (Fig 4A and 4B). In line with this finding the pro-inflammatory and neutrophil-recruiting chemokine CXCL2 as well as the pro-inflammatory cytokine IL-6 were greatly increased in setup C (Fig 4C/D). Accordingly, the numbers of recruited neutrophils increased significantly under the conditions of setup C (Fig 3B). It is of note that with none of the setups thermal injury to the fur, muscle or kidney as it may occur with the use of a heating pad could be observed. Taken together, a better control of body core temperature during experimental IR is associated with more severe kidney injury.

### Heating chamber-based optimized temperature control allows precise induction of kidney injury

**Fig 5:**
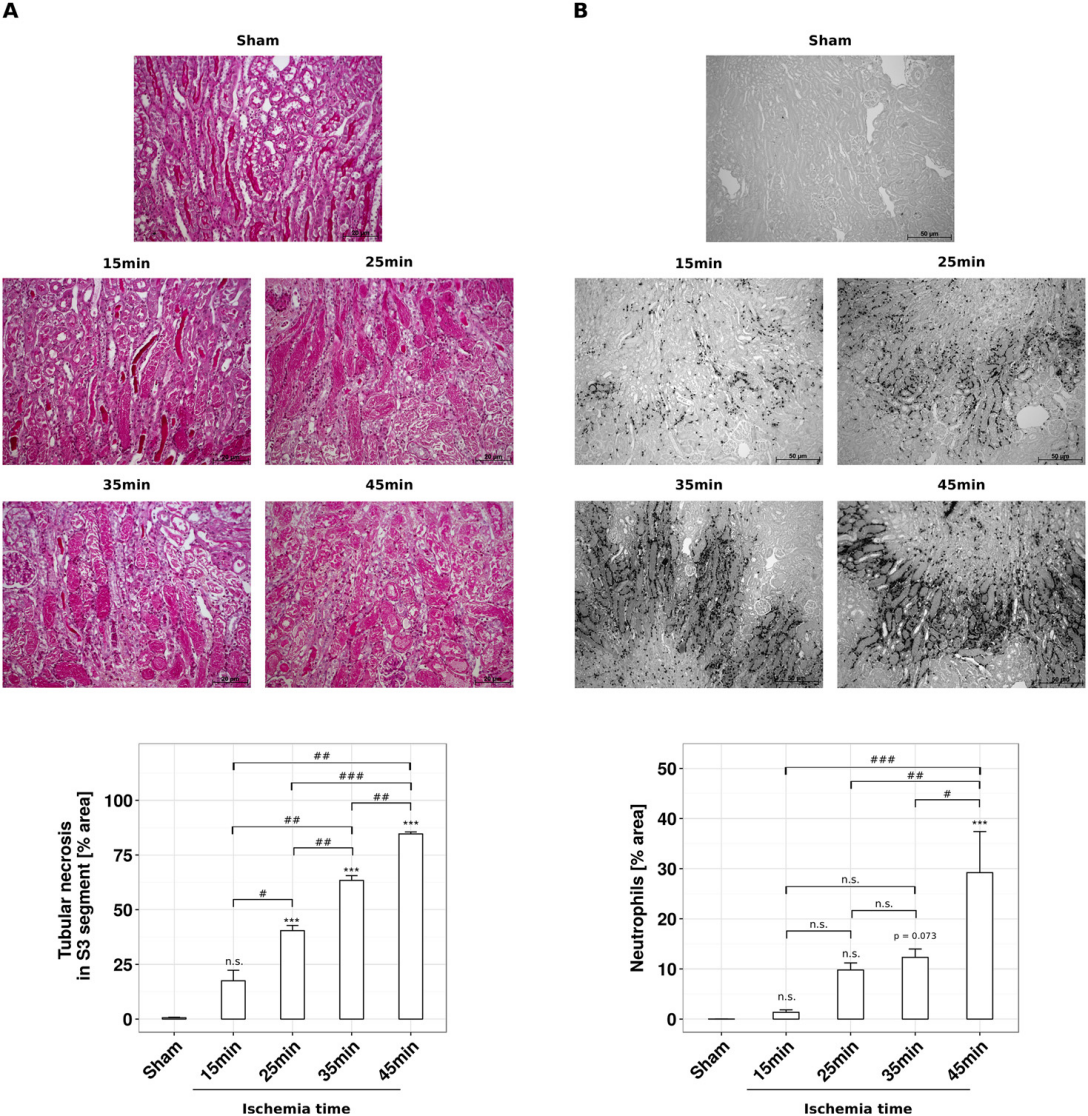
Heating chamber-based optimization of temperature control enables precise modulation of tubular injury and neutrophil infiltration at different ischemia times. Male C57BL/6N mice, 6–8 weeks of age, underwent sham surgery or unilateral ischemia for 15, 25, 35 or 45 minutes and subsequent reperfusion for 24 hours using setup C. (A) Representative PAS-stained pictures of sham-operated and ischemic kidney sections are shown at 100-fold magnification. The necrotic area of the S3 segment [%] is presented as mean ± SEM, derived from n = 5. (B) Representative Ly-6B.2-stained pictures of sham-operated and ischemic kidney sections are shown at 20-fold magnification. Ly-6B.2-positive events are assessed from 50-fold magnified images and normalized to whole kidney section area, expressed as mean ± SEM, derived from n = 5.

**Fig 6:**
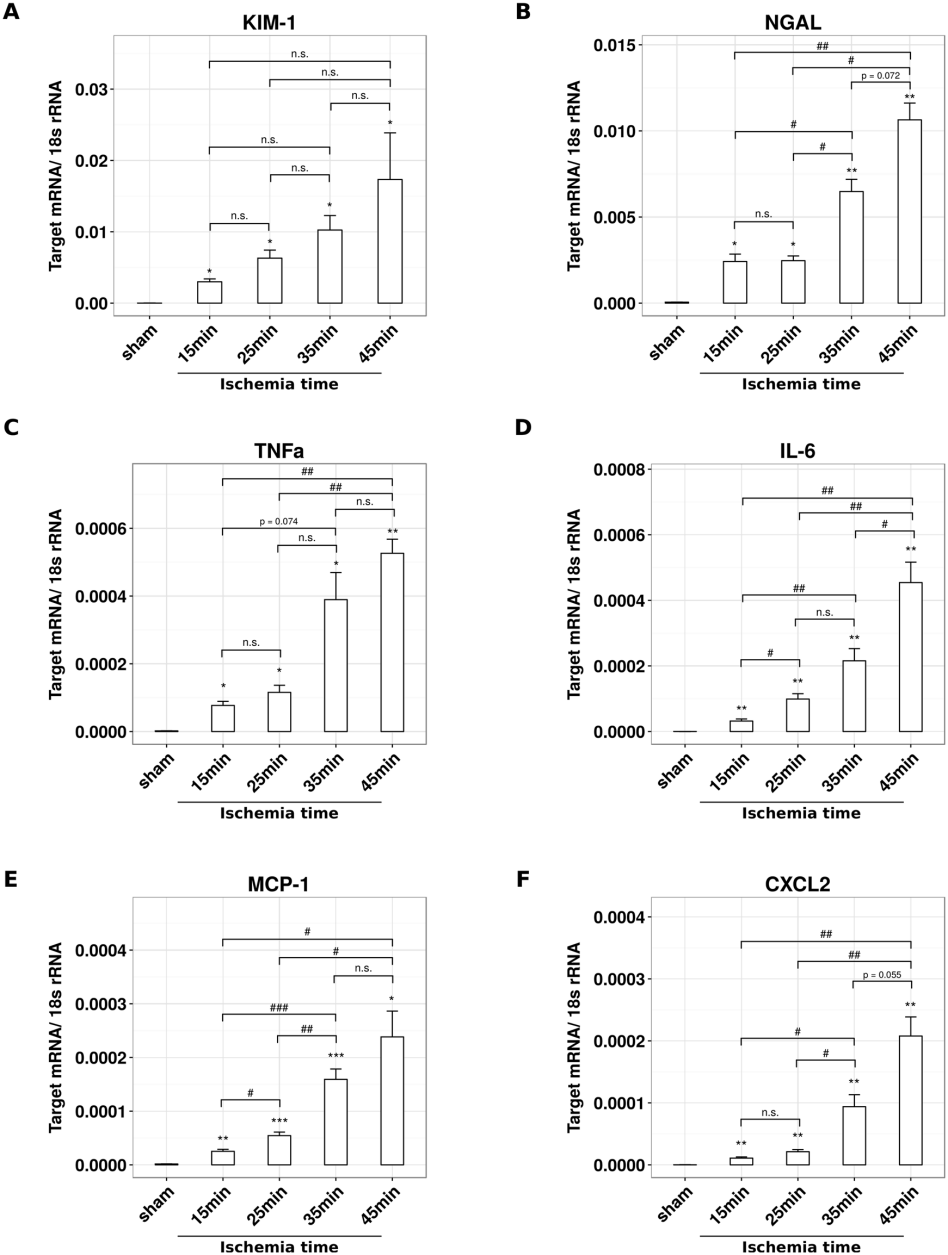
Heating chamber-based optimization of temperature control enables precise modulation of kidney injury and inflammatory marker expression by changing ischemia time. Male C57BL/6N mice, 6–8 weeks of age, underwent sham surgery or unilateral ischemia for 15, 25, 35 or 45 minutes ischemia and subsequent reperfusion for 24 hours using setup C. mRNA expression levels were assessed by reverse transcription and subsequent qRT-PCR for (A) *Kim-1*, (B) *Ngal*, (C) *TNFα*, (D) *Il-6*, (E) *MCP-1* and (F) *Cxcl-2*. Data are calculated as target gene expression normalized to the housekeeping gene 18s and presented as mean ± SEM, derived from n ≥ 5.

A better temperature control might be useful for inducing different degrees of renal IRI in a more precise manner. To prove this concept we subjected mice to either 15, 25, 35 or 45 minutes of ischemia time and 24 hours of reperfusion using setup C. Scoring necrotic lesions of the S3 segment in PAS-stained kidney sections displayed a linear increase of tubule necrosis with ischemia time at a consistently low variability (Fig 5A). This result was associated with a linear increase in neutrophil infiltrates around the injured tubules (Fig 5B). We further investigated mRNA expression profiles of injury markers and inflammatory markers by qRT-PCR (Fig 6). The levels of all investigated parameters increased with higher ischemia time, albeit not always at lower variabilities.

### Kidney atrophy after experimental kidney ischemia-reperfusion injury

**Fig 7:**
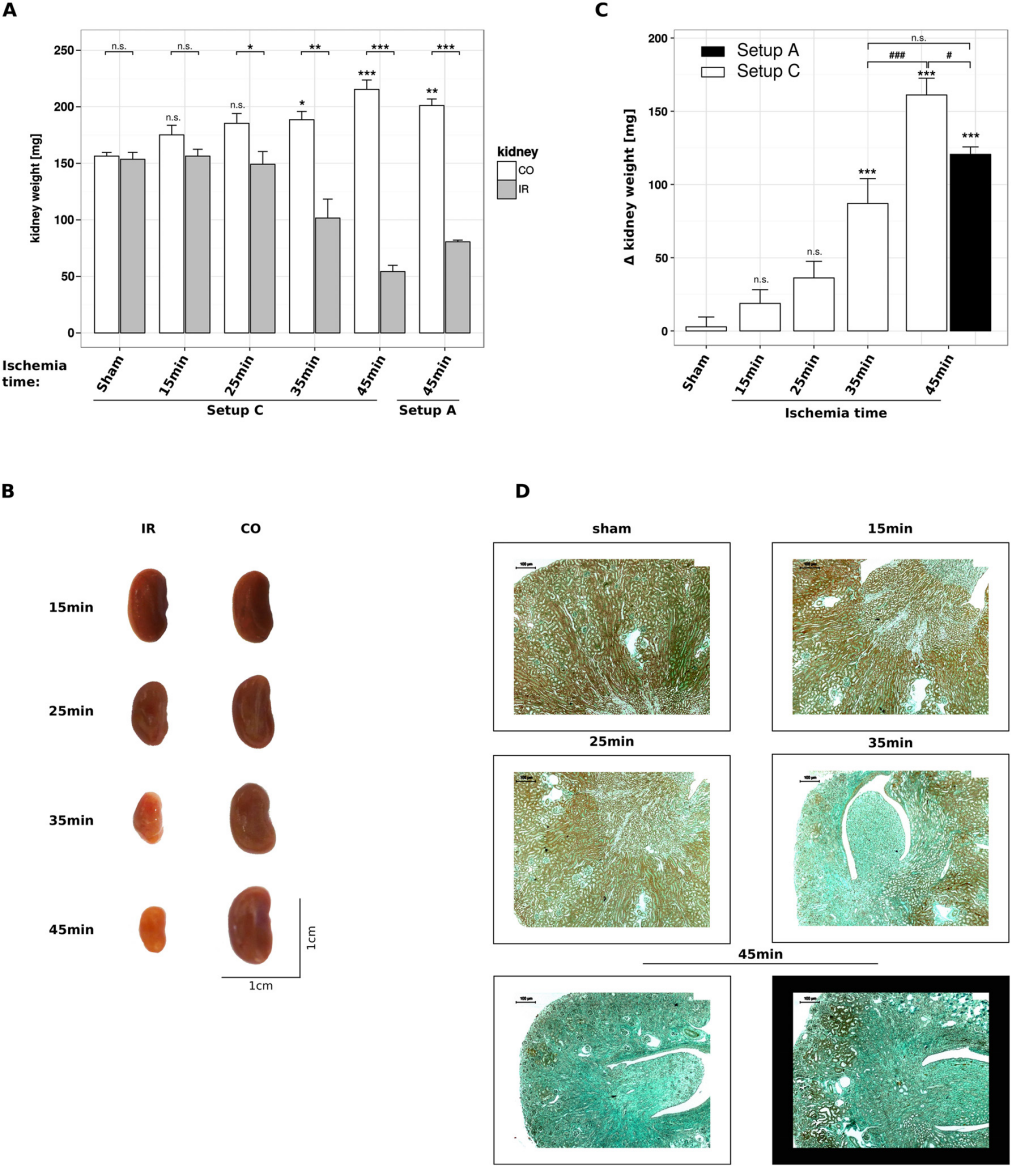
Optimizing temperature control enhances kidney atrophy after IRI. Male C57BL/6N mice, 6–8 weeks of age, underwent unilateral ischemia for 15, 25, 35 or 45 minutes ischemia and subsequent reperfusion for 5 weeks using setup A and/ or C. (A) Kidney weight [mg] is given as mean ± SEM, asteriks above CO kidneys refer to differences compared to sham-operated mice, derived from n ≥ 5. (B) Images of contra-lateral and ischemic kidneys are taken from mice that underwent IR surgery in setup C. (C) Δ kidney weight [mg] calculated from the data shown in (A) is expressed as mean ± SEM, derived from n ≥ 5. (D) Pictures of Masson’s trichrome stained kidney sections correspond to the data shown in (C).

Nephrons that do not regenerate after injury undergo atrophy and are replaced by extracellular matrix, i.e. interstitial fibrosis. The number of irreversibly lost nephrons determines the long-term outcome of an AKI episode, which often implies CKD and presents morphologically as kidney atrophy. After unilateral IRI the atrophy of the post-ischemic kidney is associated with hypertrophy of the contralateral kidney, which renders the delta (Δ) kidney weight as a very sensitive parameter of AKI-related nephron loss, kidney atrophy, and CKD [23]. Here we studied the impact of ischemia time using the temperature control protocol of setup C on AKI-related nephron loss. We used 45 minutes of unilateral ischemia with setup A as a reference point. Upon severe unilateral AKI the injured kidney underwent atrophy and the contralateral kidney increased in weight and size (Fig 7A and 7B). In sham-operated mice Δ kidney weight was negligible. Δ kidney weight increased significantly only after 35 minutes of ischemia, which indicated that lower ischemia times are associated with sufficient regeneration of the AKI-related cell loss (Fig 7C). There was no significant difference in Δ kidney weight after 35 minutes of ischemia with setup C and 45 minutes with setup A. Accordingly, 45 minutes of ischemia with setup C resulted in a significantly higher Δ kidney weight as compared to the same ischemia time with setup A (Fig 7C). Visualizing the degree of fibrotic tissue using Masson’s trichrome staining reflected these results (Fig 7D). Together, the optimized temperature control of setup C is equivalent to 10 minutes more of ischemia time in inducing IRI of the kidney.

## Discussion

We had hypothesized that online body core temperature monitoring of mice during surgical interventions can help validating temperature control. Our study confirmed this hypothesis and also shows that online body core temperature monitoring can help to optimize temperature control. Maintaining body core temperature at the physiological range reduces inter-individual variability of acute tubular necrosis following renal IRI.

Surgical procedures bear different reasons for temperature loss, which is a problem in temperature-sensitive procedures, like in IRI. While anesthesia compromises internal thermoregulation in general, the surgical wound and all surgical procedures (skin alcohol disinfection, wet gauze coverage of the exposed kidney, etc.) further enhance the temperature loss [24, 25]. With the initiation of anesthesia C57BL/6N mice with 8 weeks of age face a body core temperature reduction rate of up to 0.5°C/min (Fig 1B, setup A). Hence, it is important to take corrective action from the very first moment the body core temperature of the experimental animal starts to reduce during IRI. Our improved protocol setup C) allows for the animal to reach surgical anesthesia with efficient but mild heat supply being operative, starting from the initiation of anesthesia by i.p. injection.

Aside from controlling body temperature, maintaining good surgical conditions requires good accessibility and visibility, which—in setup C—is given during phase II and IV by using a flat heating pad illuminated by a source of bright, white light, and during phase I and III by using transparent an easy-to-use breeding chambers.

There are clearly other well established methods to control experimental animal body core temperature in the different phases of surgery. Most widely used are rectal temperature-controlled heating blanket systems, which automatically adapt the blanket’s temperature until the rodent’s rectal temperature reaches a predefined range. These systems are extremely easy-to-use but they are problematic for two important reasons:

First, heat is only supplied from the backside resulting in a temperature gradient toward the site of surgery [26]. This temperature gradient at different parts of the animal’s body is a major limitation using these systems at least in kidney research considering the kidney’s position at the flanks of the body. Therefore, direct application of heat to the fur as for example with heat pads or circulating hot-water blankets may be preferred to limit peri-surgical heat loss [9]. By using ventilated breeding devices, which are built to create and maintain a predefined micro-climate, heat supply is omni-directional which minimizes the chances of persisting temperature gradients.

Second, auto-regulated heat supply systems do not establish a stable micro-climate but correct a decline in body temperature with an increase in heat supply. In cases of severe temperature loss during IRI surgery, e.g. by midline laparotomy of several cm that is still used in some labs in contrast to a preferable 0.5–1cm flank incision, these systems are capable of increasing their temperature beyond physiologically appropriate levels, which very likely leads to hyperthermia and thermally induced necroses [26]. We observed, that mice, that are placed on a heating plate for a long time, may suffer from eye problems post-surgery, which can range from dryness to blindness, even when protecting the eyes with Bepanthene creme. In contrary, our improved protocol (setup C) delivers heat in a mild manner and within the physiological range during pre-operative care and ischemia time, and minimizes the use of a heating plate to the inevitable phases of incision/ clamping and sewing, whose duration in total does not exceed 8min per mouse in the hands of our trained surgeons. Conclusively, we did not observe any sign of necrosis, neither by hyperthermia nor by surgical stress, in any part of the kidney of sham operated mice (Figs 3–7). Hence, we believe, that our protocol addresses needs of animal wellfare in surgical models as well as the need for reduction of variance in the complex model of IRI.

It needs to be mentioned here, that our approach might precisely fit the needs of experimental IRI in kidney, but may be less appropriate for other kinds of surgical models or interventions, e.g. in vivo imaging. Still, we found the egg breeding device to be very efficient and handy in pre-operative heat supply before surgical anesthesia was reached and we expanded it’s use to all kinds of surgical interventions.

Nevertheless, every form of surgery—to a certain extent—needs to address body core temperature control as a variable. In this study we found online body core temperature monitoring to be essential for troubleshooting and protocol establishment. The high accuracy and the graphical interface of the Daisylab temperature record system used in this study allows for early recognition of trend-like deviations from the pre-defined temperature target range for every single animal (Fig 2C). Although this setting optimizes temperature control the body core temperature of few animals might escape from the target temperature range based on different susceptibilities to the narcotic drugs, different body weight/ body surface ratios or ovarian cycles in female mice [8, 15]. In these cases, online monitoring allows for controlling the effectiveness of manual interventions for every single mouse.

Beyond establishing new surgical protocols or the use of a different mouse line, sex or age in an already established surgrey, online body core temperature monitoring should be mandatory, as it serves as a tool of quality control and validation, which enables the experimentator to prove the widely used statement, that the experimental animals were kept at 37.5°C throughout the whole surgical procedure. We believe, that a more rigid body core temperature recording during experimental IRI will greatly contribute to increase reproducibility in post-ischemic injury among different laboratories [16–18].

Additionally, the use of two breeding devices and a heating plate as outlined in setup C, allows the surgeon to process up to seven mice sequentially (Fig 2C/D). Obviously, this kind of performance is only meaningful for well established temperature protocols, i.e. adapted for the mouse strain, sex and age. Nonetheless, the options to monitor and access every mouse singularly are not hindered, in case manual intervention is needed to control body core temperature due to unforeseen differences in susceptibility to the narcotic drug mixture. However, concomitant handling of seven mice with rectal probes requires attention not to cause colorectal injury.

Optimizing body core temperature control from setup A to C resulted in an improvement of inter-individual variability of tubular necrosis after renal IRI, which may allow to reduce the number of animals per group, and a mean increase of more than 3°C in body core temperature. Increasing body temperature results in more tubular damage as well as an increase in the induction of kidney injury markers [27]. Vice versa, hypothermia is long known to increase kidney tolerance to ischemia [28], which is currently used to limit organ damage in transplantation. Recently, Niemann et al. reported a reduced rate of delayed graft function in human kidney allograft recipients upon mild donor hypothermia [1]. This suggests, that in organ transplantation organ injury already starts before explantation.

Together, we conclude that online body core temperature recording can help to validate temperature control and helps to modify the experimental procedures for standardized temperature control and surgical outcomes. Based on our data we propose that online body core temperature should be monitored in each individual animal undergoing surgery.
